# Motors and Dampers: The Energetic Tradeoffs in the Shod Foot With Increasing Walking Velocity

**DOI:** 10.1002/jfa2.70101

**Published:** 2025-11-11

**Authors:** Adrienne Henderson, Dustin Bruening, Elisa Arch

**Affiliations:** ^1^ Department of Exercise Sciences Brigham Young University Provo Utah USA; ^2^ Biomechanics and Movement Science Program University of Delaware Newark Delaware USA; ^3^ Department of Kinesiology and Applied Physiology University of Delaware Newark Delaware USA

**Keywords:** gait speed, joint work, mechanical damper, midtarsal joint, shod gait

## Abstract

**Background:**

The dual influences of velocity and footwear on ankle‐foot energetics are particularly relevant for clinical populations who rely on footwear during ambulation. Although walking velocity influences energetic demands of foot structures, footwear may modify these relationships by restricting joint motion. This study aimed to characterize ankle‐foot energetics while participants walked at a wide range of velocities while wearing supportive shoes.

**Methods:**

Eighteen healthy participants walked at four height‐normalized velocities (0.4–1.0 statures/second) in supportive footwear while kinematic and kinetic data were collected. Ankle, midtarsal, and metatarsophalangeal (MTP) work was quantified and compared using repeated‐measures ANOVAs with Holm pairwise tests.

**Results:**

MTP positive and negative work increased with shod walking velocity, though negative work increased substantially more than positive work. Midtarsal positive work also increased while maintaining minimal negative work across all velocities. Ankle positive work significantly increased with velocity accompanied by small but significant increases in negative work.

**Conclusions:**

At all velocities, the MTP joint functioned as a mechanical damper and its damping characteristics became more pronounced as velocity increased. The midtarsal joint functioned as a strut, with a small motor role which became more prominent as velocity increased. The ankle had mixed roles, primarily between strut and spring, with a small damper/motor role that traded off with velocity (less damper more motor as velocity increased). The presence of supportive footwear attenuated positive and negative work across velocities when compared to previous barefoot studies, with the largest difference in the midtarsal's negative work, suggesting footwear substantially modifies natural foot mechanics through increasing velocities.

## 1 Introduction

Foot and ankle structures play a pivotal role in the energetics of walking gait and in modulating velocity, a capability that numerous clinical populations struggle to maintain. When pathologies or injuries affect these crucial structures, individuals are often unable to meet the energetic demands necessary for daily gait tasks [1, 2], leading to decreased mobility and quality of life [3]. The requirements of walking at different velocities include changing spatiotemporal parameters [4], muscle activation [4, 5, 6], muscle mechanics [7], and joint kinematics [6], kinetics [6], and energetics [8]. These relationships between velocity and gait neuromechanics have been well established for most lower extremity joints in barefoot conditions. However, footwear is often critical for patients and can have a substantial influence on ankle‐foot mechanics [9, 10]. A clear understanding of how shod ankle‐foot mechanics adapt to varying walking speeds will enhance our ability to assess and improve treatments for patients with ankle‐foot impairments.

The limited current research on barefoot ankle‐foot mechanics with varying speeds suggests that higher velocities not only increase overall energetic demand across foot structures but also change the energetic role of each foot joint. Several studies have evaluated barefoot kinematics, showing a clear increase in range of motion at both the first metatarsophalangeal (MTP) and midtarsal (combination of the calcaneonavicular and calcaneocuboid) joints as velocity increases [11, 12, 13, 14]. The single study on barefoot kinetics evaluated foot energetics at typical and maximal walking velocities [15]. The MTP joint showed an increased damper role (more negative work than positive work) and the midtarsal and ankle joints showed an increased motor role (more positive work than negative work) with the maximal walking velocity [15]. These joint changes are likely tied to both active [16] (i.e., foot musculature) and passive [17, 18, 19] (e.g., plantar fascia and elastic tendons) foot structures. Although prior research provides an initial understanding of barefoot mechanics with increasing velocities, the dual influences of footwear and gait velocity (including slow velocities) remain unclear. Addressing this knowledge gap is particularly relevant for numerous clinical populations with distal neuromuscular impairments and/or individuals who rely on footwear for support and safety.

Supportive footwear commonly used for daily ambulation constrains foot motion, thus likely altering the foot's response to changing velocity demands. On a spatiotemporal level, footwear increases step length during both walking and running by allowing for an increased foot‐to‐ground angle at initial contact. Although walking, this is accomplished primarily through increased knee extension and ankle dorsiflexion [9]. The minimal research on foot kinematics during shod walking suggests that an additional increase in ankle range of motion (RoM) [9, 10, 13] in mid to late stance from footwear may be compensating for decreased midfoot (i.e., medial longitudinal arch, MLA) RoM across stance [10]. In addition, MTP joint motion is also affected, reducing late stance extension [10, 13], which has been shown to be coupled with midfoot motion [2]. Further insights into shod foot mechanics and velocity can be derived from running studies comparing athletic shoes and barefoot conditions. Consistent with walking, running in shoes reduces midfoot dorsiflexion (i.e., MLA compression) in early stance [20]. However, in contrast to walking, running in shoes decreases ankle motion [21, 22], peak ankle plantar flexion moment [23, 24], and ankle positive work [25]. The distinct differences between walking and running on ankle‐foot mechanics are likely due to the more pronounced change in foot‐to‐floor angle at initial contact when running and reinforces velocity's significant impact on shod ankle‐foot mechanics and joint coupling. There remains a critical gap in understanding how supportive footwear modulates the coordinated mechanics of ankle‐foot joints across a broad range of walking velocities, particularly those common among clinical populations.

The purpose of this study was to characterize typical ankle‐foot joint energetics while participants walked at a wide range of velocities while wearing supportive shoes. We hypothesized that ankle, midtarsal, and MTP total stance phase positive work would significantly increase as walking velocity increased (H1). We also expected stance phase ankle negative work to significantly decrease (H2) and midtarsal and MTP negative work to significantly increase as walking velocity increased (H3). The results of this investigation have the potential to contribute to advancements in footwear design and other assistive devices that aim to optimize ankle‐foot energetics in patient populations. Furthermore, this research will facilitate the establishment of a normative reference for shod ankle‐foot energetics across a wide range of walking velocities which can be used by researchers and clinicians to distinguish between the effects attributable to an intervention, specific environment, or pathology and those resulting from variations in walking velocities.

## 2 Materials and Methods

### 2.1 Participants

Eighteen participants (10 female, 8 male, 28.61 ± 5.19 years, 76.73 ± 16.87 kg, and 1.71 ± 0.09 m) were recruited and provided their written informed consent for this study, which was approved by the University of Delaware Institutional Review Board (Approval No. 1535908‐10, Approved 02/2020). To be included in this study, participants had to be between 18 and 34 years of age, have a body mass index less than 40, and have no history of lower extremity injury or disease in the past 6 months. Potential participants were excluded if they exhibited an unsafe or unsteady gait pattern or if they had a history of systemic or lower‐extremity pathology that limited their walking ability.

### 2.2 Experimental Protocol

**FIGURE 1:**
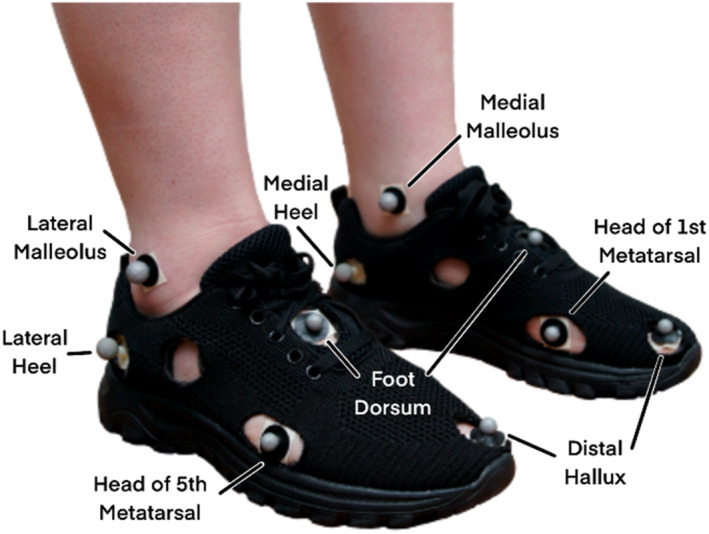
Shoes used for all speed conditions with foot markers placed.

Participants' height and weight were recorded, and they were fit with athletic‐style walking shoes to be used for the study (Figure 1). Basic, lightweight and flexible athletic shoes (Hecodi, Ningde, Fujian, China) were chosen featuring greater flexibility in the forefoot than the heel, a flat and thin foam insole, and no midfoot contour. These shoes were selected due to the prevalent use of similar styles in both healthy and clinical populations [9]. Holes were cut in the shoe uppers to allow markers to be placed directly on the feet. To preserve the structural integrity of the shoe uppers, hole sizes were minimized (approximately 5 mm larger than the marker base diameter) while still accommodating variations in foot bony anatomy at the metatarsal heads (up to ∼1 cm in the anterior/posterior direction to account for differences in toe and forefoot length). Forty‐six reflective markers were fixed to the participants for gait analysis. Anatomical markers were placed on the medial and lateral malleoli, medial and lateral femoral epicondyles, anterior superior iliac spines, posterior superior iliac spines, sternum, C7 spinous process, and both acromioclavicular joints. Clusters of four markers were placed on the bilateral thigh and shank segments. Foot markers included the following: three were placed on the heel (posterior, medial, and lateral), first metatarsal head, fifth metatarsal head, dorsum of the foot, and distal hallux (Figure 1). The navicular tuberosity and cuboid were identified using a digitizing pointer [26].

A static trial was collected before participants walked across the laboratory equipped with 12 Qualisys cameras (200 Hz, Qualisys, Gottberg, Sweden) and three AMTI force plates embedded flush with the floor (1000 Hz, AMTI Inc., Watertown, MA, USA). Participants were asked to walk at four height‐normalized walking velocities (0.4, 0.6, 0.8, and 1.0 statures/second) calculated by dividing walking speed (m/s) by participant height (m) in real time. The trials were presented in a randomized order, with 0.8 statures/sec representing a participant walking 80% of their height in distance within a second, which is often considered a typical self‐selected walking velocity [27, 28]. Practice trials were performed until participants were able to consistently achieve each new velocity. Walking velocity was monitored using a real‐time feedback program written in LabView (National Instruments, Austin, TX) [29]. Data were collected until at least three clean foot strikes for each foot were achieved while the participant walked within the targeted walking velocity (± 0.05 stat/sec) for each condition.

### 2.3 Data and Statistical Analysis

Data were first imported into Visual 3D (HAS Motion, Kingston, ON, Canada) and low‐pass filtered using a fourth‐order Butterworth filter with cutoff frequencies of 6 and 25 Hz for the motion and force data, respectively. A model was created of the torso and pelvis as well as bilateral thigh and shank segments, which were tracked using a six‐degree‐of‐freedom marker set [30]. The foot was modeled using three segments (hindfoot, midfoot, and toe) with longitudinal axes aligned parallel to the laboratory floor. The hindfoot (from the posterior heel to the midtarsal joint, defined as the midpoint between navicular and cuboid) and was tracked with the three calcaneus markers. The midfoot (midtarsal joint to the MTP joint, defined as 25% along the metatarsal head axis from the first MTP) was tracked with the cuboid, navicular, and foot dorsum markers. The toe segment (MTP joint to distal hallux) was tracked by both metatarsal heads and distal hallux markers.

Heel strike and toe‐off events for each stance phase that occurred on a force plate were placed using a 20 N minimum force threshold. Forces were assigned to each foot segment based on the anterior/posterior location of the center of pressure (COP) under the foot [31]. Briefly, the ground reaction force was assigned to the hindfoot from the instance of heel strike until the first frame in which the COP passes anterior to the midtarsal joint. At this point, the ground reaction force was assigned to the midfoot segment until the COP passed anterior to the MTP joint, at which point it was assigned to the toe segment.

Standard inverse dynamics were used to calculate joint angle, moment, and power of the ankle, midtarsal, and MTP joints during stance phase. These measures were time‐normalized to 100% stance. Moment and power were also normalized to subject mass. Positive and negative work was calculated by integrating the positive and negative portions of the power profiles, respectively. The peak moment, positive, and negative power were calculated for the ankle, midtarsal, and MTP joints. One‐way repeated measures ANOVA was used to test for significant changes in the calculated metrics between the four velocity conditions using SPSS (SPSS version 29 IBM Corporation, Armonk, NY). A Greenhouse–Geisser correction was used if data violated the assumption of sphericity. A Holm post hoc test was used for pairwise comparisons if significant main effects were observed. Significance was set to *⍺* = 0.05.

To better contextualize and compare the mechanical roles of the foot joints, we performed a secondary analysis, computing functional indices for the ankle, midtarsal, and MTP joints following the method established by Qiao et al. [32] and Kuhman et al. [33] This method quantifies the extent that a joint acts in four potential mechanical roles (damper, motor, spring, or strut) based on the joint's power and moment during the stance phase of gait, excluding any initial collision work. For the ankle and midtarsal joints, the resultant moment was used in the calculation, whereas only the sagittal plane moment was used for the one‐degree‐of‐freedom MTP joint.

## 3 Results

**FIGURE 2:**
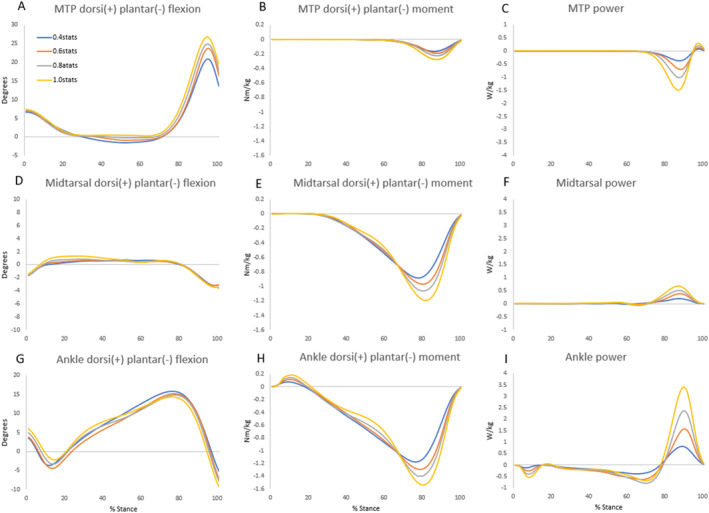
Group mean angle, moment, and power profiles for the MTP, midtarsal, and ankle joints during stance for each velocity condition. Both moment and power were normalized to subject mass.

Three foot strikes per side were collected for each participant. Metrics (means ± 1 standard deviation) along with statistical results are listed in Table 1, whereas group mean curves for each footwear condition are presented in Figure 2 for visualization. Joint indices are presented in Table 2.

**TABLE 1:** Group mean joint metrics during stance (± SD). Joint moments Nm/kg, joint peak power W/kg, and joint work J/kg.

Variable	0.4 stat/sec	0.6 stat/sec	0.8 stat/sec	1.0 stat/sec	*p*‐value
MTP					
Peak plantar flexion moment	−0.17 ± 0.05^b^ ^,^ ^c^ ^,^ ^d^	−0.19 ± 0.05^a^ ^,^ ^c^ ^,^ ^d^	−0.23 ± 0.05^a^ ^,^ ^b^ ^,^ ^d^	−0.29 ± 0.07^a^ ^,^ ^b^ ^,^ ^c^	< 0.001
Peak negative power	−0.39 ± 0.09^b^ ^,^ ^c^ ^,^ ^d^	−0.75 ± 0.21^a^ ^,^ ^c^ ^,^ ^d^	−1.08 ± 0.20^a^ ^,^ ^b^ ^,^ ^d^	−1.53 ± 0.30^a^ ^,^ ^b^ ^,^ ^c^	< 0.001
Negative work	−0.06 ± 0.02^b^ ^,^ ^c^ ^,^ ^d^	−0.08 ± 0.02^a^ ^,^ ^c^ ^,^ ^d^	−0.09 ± 0.02^a^ ^,^ ^b^ ^,^ ^d^	−0.12 ± 0.03^a^ ^,^ ^b^ ^,^ ^c^	< 0.001
Peak positive power	0.09 ± 0.03^b^ ^,^ ^c^ ^,^ ^d^	0.15 ± 0.05^a^ ^,^ ^c^ ^,^ ^d^	0.21 ± 0.06^a^ ^,^ ^b^ ^,^ ^d^	0.31 ± 0.1^a^ ^,^ ^b^ ^,^ ^c^	< 0.001
Positive work	0.005 ± 0.002^d^	0.005 ± 0.002^d^	0.006 ± 0.002^d^	0.008 ± 0.003^a^ ^,^ ^b^ ^,^ ^c^	< 0.001
Midtarsal					
Peak plantar flexion moment	−0.89 ± 0.1^b^ ^,^ ^c^ ^,^ ^d^	−0.98 ± 0.11^a^ ^,^ ^c^ ^,^ ^d^	−1.09 ± 0.11^a^ ^,^ ^b^ ^,^ ^d^	−1.20 ± 0.17^a^ ^,^ ^b^ ^,^ ^c^	< 0.001
Peak negative power	−0.05 ± 0.04	−0.06 ± 0.06	−0.09 ± 0.09	−0.09 ± 0.07	0.056
Negative work	−0.012 ± 0.010	−0.010 ± 0.008	−0.018 ± 0.006	−0.009 ± 0.005	0.072
Peak positive power	0.24 ± 0.15^b^ ^,^ ^c^ ^,^ ^d^	0.39 ± 0.14^a^ ^,^ ^c^ ^,^ ^d^	0.56 ± 0.22^a^ ^,^ ^b^ ^,^ ^d^	0.71 ± 0.32^a^ ^,^ ^b^ ^,^ ^c^	< 0.001
Positive work	0.050 ± 0.020^c^ ^,^ ^d^	0.055 ± 0.016^c^ ^,^ ^d^	0.062 ± 0.018^a^ ^,^ ^b^ ^,^ ^d^	0.072 ± 0.030^a^ ^,^ ^b^ ^,^ ^c^	< 0.001
Ankle					
Peak plantar flexion moment	−1.19 ± 0.11^b^ ^,^ ^c^ ^,^ ^d^	−1.31 ± 0.14^a^ ^,^ ^c^ ^,^ ^d^	−1.43 ± 0.13^a^ ^,^ ^b^ ^,^ ^d^	−1.55 ± 0.19^a^ ^,^ ^b^ ^,^ ^c^	< 0.001
Peak negative power	−0.45 ± 0.10^b^ ^,^ ^c^ ^,^ ^d^	−0.69 ± 0.15^a^ ^,^ ^d^	−0.90 ± 0.31^a^	−0.85 ± 0.30^a^ ^,^ ^b^	< 0.001
Negative work	−0.18 ± 0.04^d^	−0.19 ± 0.05^d^	−0.18 ± 0.04^d^	−0.15 ± 0.04^a^ ^,^ ^b^ ^,^ ^c^	< 0.001
Peak positive power	0.84 ± 0.17^b^ ^,^ ^c^ ^,^ ^d^	1.67 ± 0.34^a^ ^,^ ^c^ ^,^ ^d^	2.50 ± 0.51^a^ ^,^ ^b^ ^,^ ^d^	3.53 ± 0.71^a^ ^,^ ^b^ ^,^ ^c^	< 0.001
Positive work	0.14 ± 0.04^b^ ^,^ ^c^ ^,^ ^d^	0.17 ± 0.03^a^ ^,^ ^c^ ^,^ ^d^	0.21 ± 0.05^a^ ^,^ ^b^ ^,^ ^d^	0.27 ± 0.07^a^ ^,^ ^b^ ^,^ ^c^	< 0.001

*Note:* Pairwise comparison results.

^a^ Pairwise difference from 0.4 stat/sec.

^b^ Pairwise difference from 0.6 stat/sec.

^c^ Pairwise difference from 0.8 stat/sec.

^d^ Pairwise difference from 1.0 stat/sec.

**TABLE 2:** Group mean joint functional indices during stance.

	0.4 stat/s	0.6 stat/s	0.8 stat/s	1.0 stat/s
MTP				
Damper	51.0	54.0	58.0	62.9
Motor	0.0	0.0	0.0	0.0
Spring	8.7	8.7	8.6	9.8
Strut	39.8	36.9	33.3	26.6
Midtarsal				
Damper	0.0	0.0	0.0	0.0
Motor	7.7	8.6	9.6	11.0
Spring	5.3	4.1	3.4	2.9
Strut	87.0	87.3	86.3	86.1
Ankle				
Damper	7.6	4.4	1.5	0.0
Motor	1.5	3.0	7.6	19.0
Spring	38.4	42.6	42.1	34.2
Strut	52.5	50.1	48.8	46.7

### 3.1 MTP Joint

As walking velocity increased, peak dorsiflexion angle at the MTP joint also increased, resulting in an overall greater range of motion at this joint (Figure 2A). There was also a decrease in MTP plantar flexion during midstance with increasing velocity. The peak plantar flexion moment increased (*p* < 0.001) with each increase in walking velocity (Figure 2B, Table 1). Similarly, increased peak negative power (*p* < 0.001) and negative work (H3, *p* < 0.001) were observed (Figure 2C). Although the magnitudes were small, there was a significant increase in peak positive power (*p* < 0.001) with each increase in velocity. Positive work at the MTP joint also increased (H1, *p* < 0.001); however, there was only a significant difference between the 1.0 stat/sec velocity compared to the three slower velocities (each *p* < 0.001). As velocity increased from 0.4 to 1.0 stat/sec, the MTP joint's damper index rose from 51.0 to 62.9, whereas its secondary strut index declined from 39.8 to 26.6 (Table 2). The MTP joint showed no motor role and only a small spring index that was similar across velocities.

### 3.2 Midtarsal Joint

Midtarsal dorsiflexion increased during loading response (Figure 2D). The peak plantar flexion moment also showed a significant increase (*p* < 0.001) with each increase in walking velocity (Figure 2E, Table 1). There was no change in negative peak power or negative work at the midtarsal joint between walking conditions (H3). However, there was a significant increase in the positive peak power (*p* < 0.001) between each condition as well as an increase in positive work (*p* < 0.001) for all pairwise comparisons except when comparing 0.4 stat/sec to 0.6 stat/sec (*p* = 0.398). As velocity increased from 0.4 to 1.0 stat/sec, the midtarsal joint's modest motor index rose from 7.7 to 11.0, whereas its minimal spring index declined from 5.3 to 2.9 (Table 2). The damper index remained near zero, whereas the strut index remained high (87.0–86.1) across velocities.

### 3.3 Ankle Joint

There were no notable differences in the ankle angle between conditions except in the plantar flexion angle at toe‐off (Figure 2G), which increased with increasing velocity. Peak plantar flexion moment at the ankle significantly increased (*p* < 0.001) with each increase in walking velocity (Figure 2H). Although ankle negative work and peak negative power showed significant main effects (*p* < 0.001), the differences between conditions did not follow the order of increasing velocity (H2, Figure 2I). The 0.4 stat/sec condition had the smallest peak negative power whereas the 0.8 stat/sec had the greatest peak. A significant increase was observed in the positive peak power (*p* < 0.001) with each increase in velocity as well as an increase in positive work (*p* < 0.001). As velocity increased from 0.4 to 1.0 stat/sec, the ankle's strut index decreased slightly from 52.5 to 46.7 along with its small damper index (7.6–0.0). The ankle's motor index rose from 1.5 to 19.0 across the same velocities. The spring index remained fairly constant, increasing slightly from 38.4 to 42.6 between 0.4 and 0.6 stat/sec but then decreasing from 42.1 at 0.8 stat/sec to 32.2 at 1.0 stat/sec (Table 2).

## 4 Discussion

The primary objective of this study was to quantify the changes in ankle‐foot joint energetics during shod walking at increasing velocities. Our findings revealed negligible midtarsal negative work in shod gait, regardless of speed, in contrast to barefoot gait [15]. Similar to barefoot gait, MTP negative work and ankle, midtarsal, and MTP positive work increased with faster gait velocities [15]. Although data on barefoot gait were not collected in this study, some comparisons can be made to barefoot kinematic studies across velocities [11, 13], barefoot kinetic studies at typical velocities [2, 13, 34, 35], and a single kinetic study across velocities [15]. To facilitate comparisons with these past studies, our group mean velocities can be estimated as 0.68 ± 0.04 m/s, 1.02 ± 0.06 m/s, 1.37 ± 0.07 m/s, and 1.71 ± 0.09 m/s.

### 4.1 MTP

We observed a clear increase in MTP dorsiflexion in terminal stance with faster velocities matching Dubbeldam et al. [11] but not Sun et al. [13]. In addition, we observed a significant rise in the peak plantar flexion moment with increasing velocity. These changes contributed to the increased peak negative power and negative work (partially supports H3). At all velocities, the MTP joint showed a larger amount of negative work compared to positive work. These findings along with the high damper index support the conventional understanding of the MTP joint as the foot's primary energy sink during gait [16] and indicate that when considered in isolation, the MTP joint functions similar to a mechanical damper. An increasing, yet small amount of positive power was generated in the last 5% of stance with increasing velocities (supports H1). However, similar to Eerdekens et al. [15], as walking velocity increased, the ratio between negative work and positive work increased, amplifying the damping role of the MTP at higher velocities when shod.

When compared to previous barefoot studies [2, 15, 34, 35], we note a substantial increase in negative work and peak power with the footwear used in the present study. This appears to be driven primarily by an increased plantarflexor moment, which was also substantially higher than comparable barefoot studies, as added stiffness to the foot pushes the center of pressure more anterior throughout stance [7, 36]. This appeared to have a greater effect on MTP power than MTP motion as the plantarflexion range‐of‐motion (and likely angular velocity) was decreased compared to barefoot. We also note a possible slight decrease in peak positive power and work at our higher speeds compared to Eerdekens [15]. Overall, these comparisons suggest that shoes influence the MTP joint to act more as a damper by both increasing energy absorption and potentially by restricting the potential for subsequent energy return or generation.

### 4.2 Midtarsal

The midtarsal joint has been characterized as a spring‐like mechanism during gait, wherein the arch flattens during weight acceptance, storing mechanical energy in the soft tissues of the foot [37]. As the foot becomes unloaded during stance phase, this stored energy is returned to the joint to aid in propulsion [7, 36]. However, our findings do not support this explanation of the midtarsal joint's energetic function: the spring index remained very low across walking velocities. Moreover, essentially no negative work was observed (disproving H3) and positive work increased with velocity. Eerdekens et al. also reported an increase in positive work and no difference in negative work with increased velocity when barefoot [15]. When considering the large loads and small motion experienced by the midtarsal joint, its primary role should be considered as a strut; however, as velocity increases, the midtarsal joint increasingly has a motor role, which while small may be important for velocity modulation.

Previous studies examining barefoot midtarsal motion across walking velocities have consistently demonstrated a decrease in mid‐stance dorsiflexion prior to an increase in late‐stance plantar flexion with increasing velocity [12, 13, 38], matching the shift from decreased negative to increased positive work noted above. In our study, we saw near‐zero midtarsal motion across most of stance. Thus, despite an increased midtarsal plantarflexion moment, there was also very little negative power across velocities. We did note a few degrees of plantarflexion in late stance along with somewhat similar positive work compared to barefoot studies [2, 15, 34, 35]. Overall, these comparisons suggest that shoes, in markedly restricting midtarsal dorsiflexion, shift the midtarsal to be more motor‐like.

Recent barefoot studies indicate that there is a kinetic link between the MTP and midtarsal joints [2, 17]. These previous investigations have demonstrated that when motion at the MTP joint was restricted, the expected decrease in MTP negative work was accompanied by a concomitant decrease in midtarsal motion and positive power/work [2]. Similarly, in the present study, the increase in MTP negative work that occurred with faster walking velocities was accompanied by a slightly smaller increase in midtarsal positive work. In other words, as velocity increased, the MTP joint became more damper‐like, whereas the midtarsal joint became slightly more motor‐like. This energetic connection between the foot joints could be highly efficient if it were a passive energy transfer from the toes to the midtarsal joint as described by an inextensible windlass mechanism. Our results add to prior work [17, 39, 40] suggesting that this mechanism does not appear to be a significant contributor to shod foot energetics, as there was no substantial change in midtarsal plantar flexion motion with increased toe dorsiflexion. More likely, the biarticular muscles that cross both the midtarsal and MTP joints, such as the flexor digitorum brevis, flexor hallucis brevis, and quadratus plantae (via the tendons of flexor digitorum longus), play an active role in the increased midtarsal positive power.

### 4.3 Ankle

The ankle also exhibited increasing motor‐like behavior with increasing velocity, displaying a substantial increase in its motor index and significant increases in positive work with a slight decrease in negative work at the fastest velocity (supporting H2). Past barefoot studies show similar trends, with an increased motor index [33], slightly greater decreases in negative work and similar increases in positive work as velocity increases [4, 15]. The increased motor‐like role of the ankle is also consistent with studies that showed increased muscle activation as participants walked faster [4, 5, 41]. Since both the ankle and midtarsal joints become more motor‐like with increased walking velocity, the biarticular muscles that cross both joints (tibialis anterior, tibialis posterior, and fibularis longus) may have comparable influences on both joints' role in achieving the faster velocities. In addition, two muscles (flexor hallucis longus and the flexor digitorum longus) cross all three of the ankle, midtarsal, and MTP joints. As gait velocity increases and the MTP increases its dorsiflexion angle, the ankle and midtarsal joints may work together to minimize length changes in these muscles (i.e., force‐length and force‐velocity relationships). Future research is needed to confirm the theory that this active muscular coupling between the three joints may play a more significant role in velocity modulation than any passive coupling mechanism.

### 4.4 Limitations and Future Research

Including a barefoot condition would have allowed us to more directly compare the effects of the shoes on foot and ankle energetics. However, doing this would have effectively doubled the already high number of trials needed for analysis. Thus, we opted for a comparison to prior studies. We also only investigated a single type of shoe for similar reasons. Future studies should investigate if the findings from the present study are consistent with other types of footwear and footwear designs, especially footwear with and without orthoses commonly found in clinical populations. These studies could help determine whether certain footwear features or orthotic devices can assist the ankle and foot joints in achieving their typical energetic function as seen in barefoot gait. Additionally, future studies may benefit from measuring participants' natural foot posture and foot muscle strength as both factors may influence gait function. A larger number of participants could allow future researchers the ability to analyze subgroups based on foot posture or strength differences or on other characteristics (e.g., sex).

### 4.5 Conclusions and Clinical Implications

Overall, these results emphasize (1) the increasing energetic demand of the ankle and foot joints with increasing walking velocities and (2) the restrictive impact of footwear on these roles. As walking velocity increases, the ankle and midtarsal joints become less damper, strut, and spring‐like and more motor‐like, thus any soft tissues that store and return energy at these joints during gait contribute a reduced proportion of energy while active muscle contractions contribute a larger proportion. This finding is particularly significant when considering footwear design, passive‐dynamic ankle‐foot orthoses, and active prosthetic devices. The design of passive‐dynamic ankle‐foot orthoses mirrors the spring‐like behavior exhibited by the ankle and midfoot during slower walking velocities [42], as it stores energy during midstance and then returns it to the biological system during propulsion. Although passive biological structures and external devices likely continue to play a role in gait energetics regardless of walking velocity, the increasing motor‐like role of the ankle and midfoot at faster velocities may reduce the impact of these passive structures as well as assistive devices [7]. Consequently, this may diminish an individual's ability to efficiently increase their walking velocity. When using a powered prosthetic ankle‐foot or exoskeleton, increasing walking velocity is typically achieved by increasing the power output at the ankle. This approach overlooks the midfoot's motor‐like contribution to increasing gait velocity as well as its influence over the repositioning of the ankle and foot joints for enhanced push‐off mechanics [43]. The findings from this study provide critical insights that can influence the design of future footwear and assistive devices through implementing aspects that account for interconnected ankle, midfoot, and MTP velocity‐dependent energetic contributions to increasing gait velocities. Moreover, this analysis establishes a normative reference of shod ankle‐foot energetics across walking velocities that can serve as a baseline for distinguishing velocity‐dependent effects from intervention outcomes or pathological conditions.

## Author Contributions

**Adrienne Henderson:** conceptualization, formal analysis, investigation, methodology, project administration, resources, software, visualization, writing – original draft, writing – review and editing. **Dustin Bruening:** formal analysis, funding acquisition, methodology, software, visualization, writing – review and editing. **Elisa Arch:** conceptualization, formal analysis, funding acquisition, methodology, resources, supervision, writing – review and editing.

## Ethics Statement

This study was approved by the University of Delaware Institutional Review Board, Approval No. 1535908‐10.

## Conflicts of Interest

The authors declare no conflicts of interest.

## Data Availability

The motion and force data used to support the findings of this study are available from the corresponding author upon reasonable request.
